# Real-world long-term efficacy and safety of erenumab in adults with chronic migraine: a 52-week, single-center, prospective, observational study

**DOI:** 10.1186/s10194-022-01433-9

**Published:** 2022-06-02

**Authors:** Christopher Kjaer Cullum, Thien Phu Do, Messoud Ashina, Lars Bendtsen, Sarah Sonja Hugger, Afrim Iljazi, Julia Gusatovic, Josefin Snellman, Cristina Lopez-Lopez, Håkan Ashina, Faisal Mohammad Amin

**Affiliations:** 1 Danish Headache Center, Department of Neurology, Rigshospitalet Glostrup, Faculty of Health and Medical Sciences, University of Copenhagen, Copenhagen, Denmark; 2 Novartis Pharma AG, 4033 Basel, Switzerland; 3 Roche Innovation Center Basel, F. Hoffmann-La Roche Ltd Grenzacherstrasse 124, Basel, Switzerland; 4 Department of Neurorehabilitation / Traumatic Brain Injury, Rigshospitalet, Copenhagen, Denmark

**Keywords:** Chronic migraine, Erenumab, Preventive treatment, Calcitonin gene-related peptide, Headache, mAb, Adverse events

## Abstract

**Background:**

Clinical trials have shown that erenumab is effective and well-tolerated for the preventive treatment of chronic migraine. To extend the results from clinical trials, we assessed the real-world efficacy and safety of erenumab in patients with chronic migraine from the outpatient clinic at the Danish Headache Center.

**Methods:**

A 52-week, single-center, prospective, observation study of erenumab in adults with chronic migraine who are eligible for treatment with monoclonal antibodies against CGRP or its receptor in Denmark. The primary outcome was defined as proportion of patients who achieved ≥ 30% reduction in monthly migraine days (MMDs) from baseline to weeks 9–12.

**Results:**

A total of 300 adult patients with chronic migraine were enrolled and received at least one dose of erenumab. At baseline, the mean (SD) number of monthly headache days was 23 ± 4.9 and mean number of MMDs was 16.8 ± 6.4. Of 300 enrolled patients, 273 (91.0%) patients completed 12 weeks of treatment, and 119 (39.7%) completed 52 weeks of treatment. The number of patients who achieved ≥ 30% reduction in MMDs from baseline to weeks 9–12 was 195 (71.4%) of 273 patients. Sustained ≥ 30% reduction in MMDs at all assessment periods throughout the 52-week treatment period was achieved by 102 (34%) of 300 patients. Adverse events occurred in 220 (73.3%) out of 300 patients. The most common adverse event was constipation. Treatment discontinuation due to lack of tolerability occurred in 41 (13.7%) patients.

**Conclusions:**

Among adult patients with chronic migraine and previous failure of medications for migraine prevention, erenumab was found to be effective and well-tolerated.

## Introduction

Migraine is a disabling neurologic disorder that afflicts more than one billion people worldwide [1, 2]. According to the Global Burden of Disease Study [3], migraine is a leading cause of years lived with disability worldwide. The attributable burden is highest in those affected by chronic migraine for whom effective management often, if not always, requires treatment with preventive medications [2, 4, 5]. The availability of medications targeting calcitonin gene-related peptide (CGRP) or its receptor has recently expanded our therapeutic armamentarium and holds promise to address unmet treatment needs [4].

Erenumab is a fully human monoclonal antibody (mAb) against the CGRP receptor that has been approved for the preventive treatment of migraine in adults, including chronic migraine [6, 7]. Its long-term efficacy and safety profile has been confirmed in a 52-week open-label extension study following a 12-week double-blind phase [8]. However, randomized controlled trials (RCTs) include carefully selected study populations, and real-world data is consequently considered valuable to generate evidence from routine clinical practice [4]. The present study assessed the long-term efficacy and safety of erenumab in a 52-week, single-center, prospective, observational study that includes adult patients with chronic migraine who are eligible for treatment with mAbs against CGRP or its receptor in Denmark.

## Methods

### Study oversight

The present study was approved by the relevant ethics committee and the Danish Data Protection Agency. All enrolled patients provided written informed consent before any study-related tasks or procedures were performed. The study was conducted in accordance with the principles of the Declaration of Helsinki [9].

### Study design and procedures

A single-center, prospective, 52-week observational study of erenumab for adults with chronic migraine. The study included a 4-week run-in period and a 52-week treatment period. There were six scheduled visits at the outpatient clinic of the Danish Headache Center: screening, baseline (1^st^ dose), week 12, week 24, week 36, and week 52 (final evaluation). Patients were instructed to fill out a headache diary (paper format) with daily entries throughout the study. To receive treatment with erenumab, patients were required to present with a complete 4-week headache diary at the baseline visit. The diary was used to record information on the frequency of migraine and headache, as well as to capture data on adverse events. During study visits, study personnel reviewed the diary entries and entered the data in the patients’ electronic medical records.

According to local practice guidelines, patients would receive 140-mg of erenumab every 4 weeks (28 days) from baseline to week 12. At the baseline visit, site personnel instructed patients on proper technique for self-administration of erenumab and supervised injection of first dose to ensure correct administration. All subsequent doses were conducted as self-administrations by the patients. Efficacy was then evaluated at the week 12 visit and patients who experienced < 30% reduction in monthly migraine days (MMDs) from baseline to week 9 through 12 were classified as non-responders and discontinued treatment. Patients who were classified as responders would then continue treatment for the following 12 weeks (i.e., week 13 through 24), with a reduced dose of 70-mg. At the week 24 visit, the treating physician would assess whether a greater reduction in MMDs or better tolerability had been achieved with 140-mg or 70-mg erenumab. Based on this information and patient preference, the treating physician would decide if patients were to continue with 70-mg erenumab or receive an increased dose of 140-mg erenumab until study end (i.e., week 52). This assessment was only performed once at the week 24 study visit with no further dose modifications.

### Study population

Adult patients were eligible for inclusion if they had a diagnosis of chronic migraine in accordance with the International Classification of Headache Disorders, 3^rd^ edition (ICHD-3) [10]. Entry criteria also included documented failure based on lack of efficacy or tolerability of at least one antihypertensive and one anticonvulsant that is used for migraine prevention. [2, 4, 5, 11]. Exclusion criteria were a diagnosis of medication-overuse headache (MOH) at the time of study enrollment, as defined in ICHD-3. [10]. Female patients were also excluded if they were pregnant, planning to become pregnant, or lactating. These eligibility criteria had been defined by regulatory authorities in Denmark. All patients who were scheduled to initiate treatment with erenumab from January 2019 were approached for inclusion until a total of 300 patients who received at least one dose of erenumab were included.

### Outcomes

The primary outcome was defined as the proportion of patients who achieved ≥ 30% reduction in MMDs from baseline to weeks 9–12. The secondary outcome was defined as the proportion of patients who achieved ≥ 50% reduction in MMDs from baseline to weeks 9–12. Exploratory efficacy outcomes included: (1) the proportion of patients who achieved ≥ 75% reduction in MMDs from baseline to weeks 9–12, (2) the absolute change in MMD from baseline to weeks 9–12, (3) the absolute change in monthly headache days (MHDs) from baseline to weeks 9–12, (4) the proportion of patients who converted from chronic to episodic migraine from baseline to weeks 1–12, (5) the absolute change in mean MMD from baseline to weeks 13–24, (6) the absolute change in mean MHD from baseline to weeks 13–24 (7) the proportion of patients who achieved ≥ 30% reduction in mean MMDs from baseline to weeks 41–52 on 140-mg erenumab, (8) and the proportion of patients who achieved ≥ 30% reduction in mean MMDs from baseline to weeks 41–52 on 70-mg erenumab, (9) the absolute change in mean MMD from baseline to weeks 41–52, (10) the absolute change in mean MHD from baseline to weeks 41–52, (11) the proportion of patients who converted from chronic to episodic migraine from baseline to weeks 41–52.

### Statistical analysis

Outcomes were calculated based on headache diary entries. All continuous or categorial variables are reported as mean with either 95% confidence interval (95% CI) or standard deviations (SD); if data had a skewed distribution, median with interquartile range were reported. Responder rates are reported as percentages. Except for weeks 9–12, MMDs and MHDs were calculated as a monthly mean across the assessment period, i.e., weeks 13–24, weeks 25–36, and weeks 41–52. We conducted a complete case analysis of efficacy data, which were reported as observed. Adverse events were reported for all patients who received at least one dose of erenumab. SPSS Statistics version 27.0 for Mac was used for statistical analyses.

## Results

**Fig. 1:**
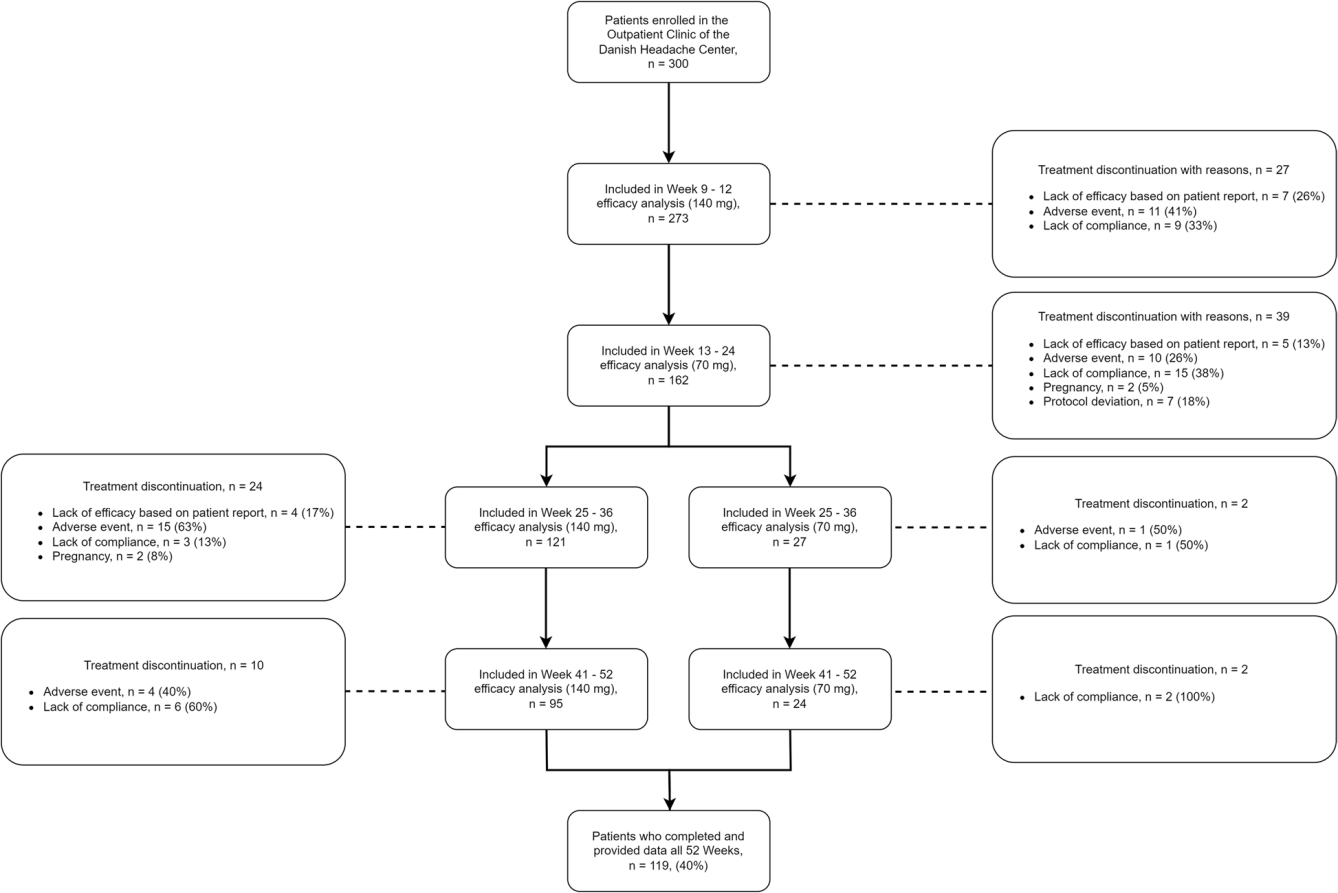
Patient Flowchart and Disposition. All patients were started on 140-mg erenumab and received this dose from week 1 through week 12. After the initial 12 weeks of treatment, all patients had a dose reduction and received 70 mg erenumab from week 13 through week 24. From week 24 through week 52, patients continued with the dose with the highest efficacy (140-mg or 70-mg). Final evaluation was conducted at week 52

A total of 300 adult patients with chronic migraine received at least one dose of erenumab at the outpatient clinic of the Danish Headache Center between January 2019 and February 2020. Of these, 273 (91%) patients completed 12 weeks of treatment, and 119 (39.7%) completed 52 weeks of treatment (Fig. 1). The study population had a mean age of 45.9 ± 13.1 years, and 257 (75.7%) of 300 patients were women. The baseline mean MHDs was 23.0 ± 4.9 days, and the baseline mean MMDs was 16.8 ± 6.4 days. All patients had discontinued their use of at least two preventive medications for migraine due to lack of efficacy or tolerability, as documented in medical records. The median number of previously failed migraine preventive medications was 7 (interquartile range: 5–9). Overall, 282 (94%) of 300 patients had failed ≥ 4 preventive medications, and 222 (74%) of 300 patients ≥ 6 preventive medications. Candesartan (*n* = 284, 94.7%), topiramate (*n* = 236, 78.7%), and metoprolol (*n* = 204, 68%) were the most common previous preventive medications that patients had failed. Concomitant preventive medication was used by 89 patients at the time they received their first dose of erenumab. Table 1 summarizes baseline characteristics of the study population.

**Table 1:** Baseline Characteristics of the Study Population

Study Population Characteristics	All Participants (*n* = 300)
**Age,** mean, years (SD)	45.9 (13.1)
**Women,** *n* (%)	257 (85.7%)
**Number of Monthly Headache Days,** mean, days (SD)	23 (4.9)
**Number of Monthly Migraine Days,** mean, days (SD)	16.8 (6.4)
**Number of Prior Failed Preventive Medications,** median (IQR)	7 (5 – 9)
**Number of Patients using Concomitant Preventive Medication at Baseline,** *n* (%)	89 (29.7%)

All patients were diagnosed with chronic migraine without medication overuse headache. Abbreviations: *SD* standard deviation, *IQR* interquartile range

### Efficacy

**Fig. 2:**
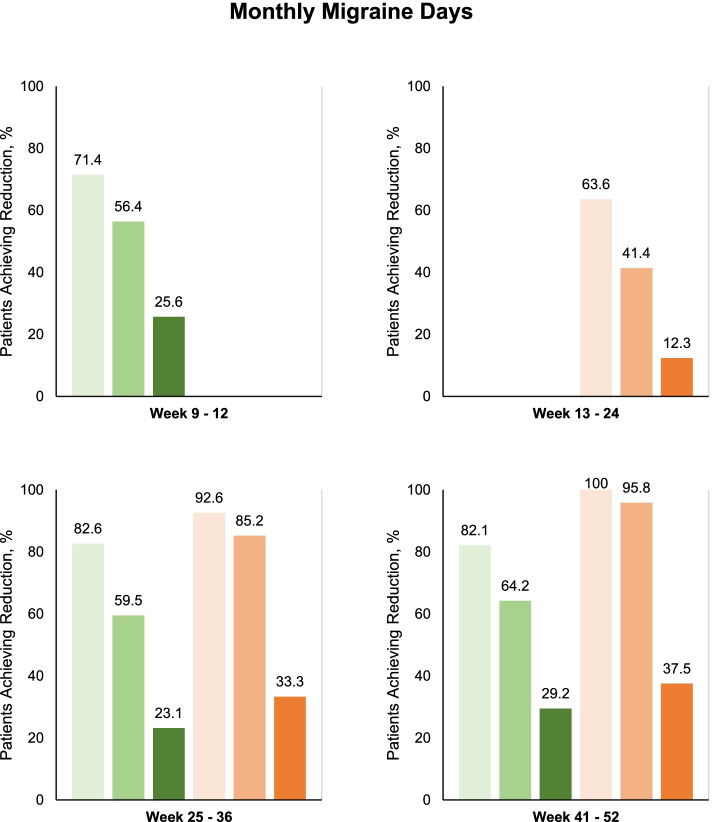
Proportion of patients with ≥ 30%, ≥ 50%, and ≥ 75% reduction in number of monthly migraine days. Green represents patients treated with 140-mg erenumab, light: ≥ 30%, medium: ≥ 50%, and dark: ≥ 75%. Orange represents patients treated with 70-mg erenumab, light: ≥ 30%, medium: ≥ 50%, and dark: ≥ 75%. Participants with data available for analysis; Weeks 9–12, *n* = 273; Weeks 13–24, *n* = 162; Weeks 25–36; 140-mg, *n* = 121; 70-mg, *n* = 27; Weeks 41–52; 140-mg, *n* = 95; 70-mg, *n* = 24

**Fig. 3:**
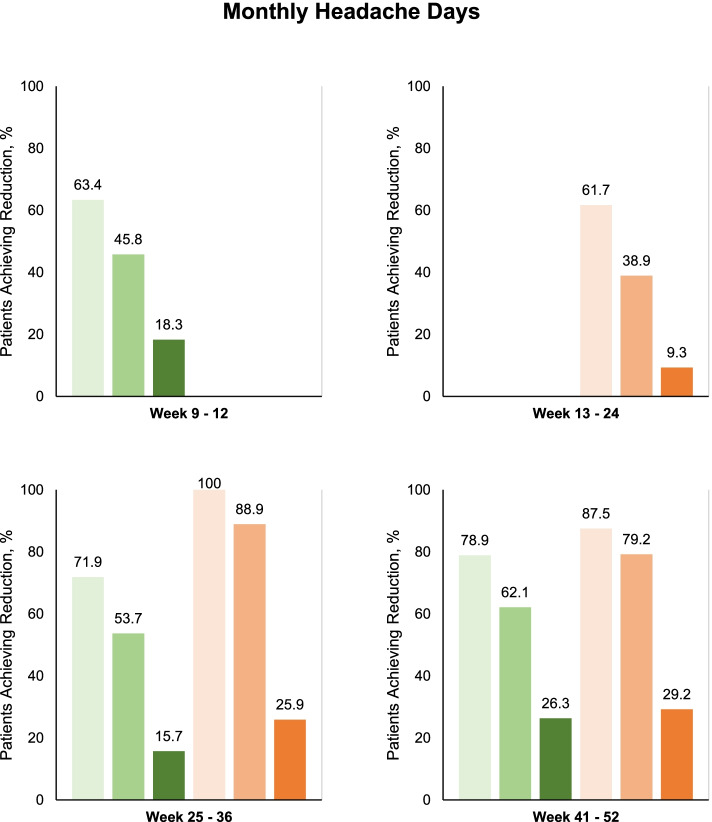
Proportion of patients With ≥ 30%, ≥ 50%, and ≥ 75% reduction in number of monthly headache days of any severity. Green represents patients treated with 140-mg erenumab, light: ≥ 30%, medium: ≥ 50%, and dark: ≥ 75%. Orange represents patients treated with 70-mg erenumab, light: ≥ 30%, medium: ≥ 50%, and dark: ≥ 75%. Participants with data available for analysis; Weeks 9–12, *n* = 273; Weeks 13–24, *n* = 162; Weeks 25–36; 140-mg, *n* = 121; 70-mg, *n* = 27; Weeks 41–52; 140-mg, *n* = 95; 70-mg, *n* = 24

The number of patients who achieved ≥ 30% reduction in MMDs from baseline to weeks 9–12 was 195 (71.4%) of 273 patients (Fig. 2). The corresponding figures were 154 (56.3%) of 273 patients for a ≥ 50% response and 70 (25.6%) of 273 patients for a ≥ 75% response (Figs. 2, 3). The change in MMDs from baseline to weeks 9–12 was -7.8 days (95% CI, -8.6 to -7.0) while the corresponding change in MHDs -8.9 days (95% CI, -9.8 to -8.1). Conversion from chronic to episodic migraine was achieved by 180 (65.9%) of 273 patients from baseline to weeks 1–12.

After 12 weeks of treatment, 195 were classified as treatment responders based on a ≥ 30% reduction in MMDs from baseline to weeks 9–12. Of these, 162 patients continued and completed treatment with a reduced dose of 70-mg erenumab from weeks 13–24. The proportion of patients achieving ≥ 30% reduction in mean MMDs from baseline to weeks 13–24 was 103 (63.6%) of 162 patients (Fig. 2). The corresponding figures were 67 (41.4%) of 162 patients for a ≥ 50% response rate in MMDs and 20 (12.3%) of 162 patients for a ≥ 75% response rate in MMDs. The change in mean MMDs from baseline to weeks 13–24 was -6.5 days (95% CI, -7.4 to -5.6) while the corresponding change in mean MHDs was -8.2 days (95% CI, -9.2 to -7.3).

At the week 24 site visit, 128 patients returned to treatment from 70-mg to the initial 140-mg erenumab while 28 patients continued on the lower 70-mg erenumab. Efficacy data from weeks 25–36 were available from 121 of 128 patients on 140-mg erenumab and 27 of 28 patients on 70-mg erenumab. The proportion of patients achieving ≥ 30% reduction in mean MMDs from baseline to weeks 25–36 was 100 (82.6%) of 121 patients on 140-mg erenumab and 25 (92.6%) of 27 patients on 70-mg erenumab. For patients on 140-mg erenumab, the change in mean MMDs from baseline to weeks 25–36 was -8.9 days (95% CI, -9.9 to -7.9) while the corresponding change in mean MHDs was -10.2 days (95% CI, -11.4 to -9.1). For patients on 70-mg erenumab, the change in mean MMDs from baseline to weeks 25 – 36 was -9.5 days (95% CI, -11.5 to -7.5) while the corresponding change in mean MHDs was -13 days (95% CI, -14.8 to -11.3).

At the week 36, visit, 100 patients on 140-mg erenumab and 25 patients on 70-mg erenumab were eligible to continue treatment with erenumab until study end, i.e., week 52. Efficacy data from weeks 41–52 were available from 95 of 100 patients on 140-mg erenumab and 24 of 25 patients on 70-mg erenumab. From baseline to weeks 41–52, the proportion of patients achieving ≥ 30% reduction in mean MMDs was 78 (82.1%) of 95 patients on 140-mg erenumab and 24 (100%) of 24 patients on 70-mg erenumab (Fig. 2); thus, a total of 102 (34%) of the initial 300 patients included had a sustained ≥ 30% reduction in mean MMDs throughout all assessment periods. The change in mean MMDs from baseline to weeks 41–52 was -9.5 days (95% CI, -10.8 to -8.2) on 140-mg erenumab and -9.3 days (95% CI, -11.1 to -7.4) on 70-mg erenumab. The change in mean MHDs from baseline to weeks 41–52 was -11.8 days (95% CI, -13.1 to -10.5) on 140-mg erenumab and -11.8 days (95% CI, -13.7 to -9.9) on 70-mg erenumab. Conversion from chronic to episodic migraine was achieved by 77 (81.1%) of 95 patients on 140 mg erenumab and by 22 (91.7%) of 24 patients on 70 mg erenumab from baseline to weeks 41–52.

### Tolerability and safety

During the 52-week treatment period, adverse events were reported by 220 (73.3%) of 300 patients who received at least one dose of erenumab. The most common adverse events were constipation (*n* = 124), nausea (*n* = 22), and fatigue (*n* = 20). Treatment discontinuation due to adverse events occurred in 41 (13.7%) patients. Serious adverse events were reported by one patient (pulmonary embolism), who did not discontinue treatment; this patient had comorbidities in the form of Addison’s disease, hypothyroidism, and obesity. Table 2 provides an overview of reported adverse events throughout the treatment period.

**Table 2:** Treatment-emergent adverse events

Event	All Participants (*n* = 300)
**Any Adverse Event, n (%)**	220 (73.3%)
**Serious Adverse Events, n (%)**	1 (0.3%)
**Adverse Events Leading to Treatment Discontinuation, *n* (%)**	41 (13.7%)
*Most Frequent Adverse Events Leading to Treatment Discontinuation	
Constipation, *n* (%)	22 (7.3%)
*Most Frequent Any Adverse Event	
Constipation, *n* (%)	124 (41.3%)
Injection site reaction, n (%)	29 (9.7%)
Nausea, *n* (%)	22 (7.3%)
Fatigue, *n* (%)	20 (6.7%)
Aggravation of migraine, n (%)	14 (4.7%)
Tinnitus, *n* (%)	14 (4.7%)
Alopecia, *n* (%)	11 (3.7%)
Muscle cramps, n (%)	11 (3.7%)
Dizziness, *n* (%)	10 (3.3%)
Abdominal pain, *n* (%)	9 (3%)
Insomnia, *n* (%)	8 (2.7%)
Metrorrhagia, *n* (%)	6 (2%)
Weight gain, *n* (%)	6 (2%)
Hot flashes, *n* (%)	6 (2%)
Flushing, *n* (%)	6 (2%)

^*^Adverse events occurring in ≥ 2% of participants

## Discussion

In this 52-week observational study from real-world clinical practice, we examined the effectiveness and safety of erenumab in adult patients with chronic migraine who are eligible for treatment with mAbs against CGRP or its receptor in Denmark. The results show that 71% of patients achieved ≥ 30% reduction in MMDs from baseline to weeks 9–12. This figure is somewhat higher than in other observational studies of 140-mg erenumab for migraine prevention [12, 13]. These studies found that 42–60% of patients with chronic migraine achieved ≥ 30% reduction in MMDs from baseline to weeks 9–12 [12, 13].

Given the design of our study, we cannot draw any firm conclusions on the effectiveness of 140-mg erenumab *versus* 70-mg erenumab. The reason is that the initial 12 weeks of treatment with 140-mg erenumab might have been influenced by placebo effect. In contrast, the subsequent 12 weeks of treatment with 70-mg erenumab is likely subject to nocebo effects since the patients knew that the dose had been reduced. Further research is needed to ascertain the long-term effectiveness and safety of 140-mg erenumab *versus* 70-mg erenumab in a real-world setting.

In view of the high number of previous preventive medication failures, it merits emphasis that 102 (34%) of 300 patients who received at least one dose of erenumab achieved at ≥ 30% reduction in MMDs throughout the 52-week treatment period. This figure is lower than in other real-world observational studies which, in part [12, 14], might be explained by the treatment discontinuation of patients who experienced less than 30% reduction at the various assessment periods (i.e., weeks 9–12, weeks 25–36). The latter principle was based on local practice guidelines. Our results mirror the use of erenumab in Denmark, where clinicians can only prescribe erenumab to adult patients with chronic migraine and documented failure of at least two medications for migraine prevention. It should also be noted that, at present, erenumab can only be prescribed by hospital-based neurologists in Denmark. Our findings therefore cannot be generalized to patients with migraine who are treated in the primary care setting or those who are diagnosed with episodic migraine. In addition, our study population did not include patients who have chronic migraine with comorbid MOH.

The frequency of adverse events in our study is higher than in clinical trials [8, 15, 16]. Similar findings have been made in other real-world studies which, in part, might be explained by more careful selection of study populations in clinical trials [12–14, 17–22]. The most common adverse event, and reason for treatment discontinuation due to lack of tolerability, was constipation. This is congruent with observations from clinical trials as well as real-world evidence [12–23]. Future observational studies should assess whether erenumab is better avoided in patients with a history of recurrent constipation. Furthermore, it would be helpful to examine whether constipation is more often a single-episode or recurrent event in patients with migraine.

### Limitations

Our study has several limitations some of which have already been outlined above. The choice of MMDs as the primary efficacy outcome is recommended in clinical practice [24]. However, the reduction in MHDs of moderate to severe intensity is usually considered a useful co-primary efficacy outcome because some patients might experience a clinically meaningful effect on the intensity rather than frequency of headaches [24]. It is reasonable to argue that both efficacy outcomes should be used to better ascertain the response to treatment in clinical practice. Moreover, it should be acknowledged that we used a headache diary with daily entries in paper format. An electronic headache diary is often preferred due to the availability of time stamp features. According to local practice guidelines, patients were excluded from treatment if they did not achieve a ≥ 30% reduction in MMDs at each assessment period; thus, reported responder rates are for those subjects who remain at each assessment period.

## Conclusions

Among adult patients with chronic migraine and previous failure of medications for migraine prevention, erenumab was found to be an effective and well-tolerated treatment. The results underscore that the therapeutic benefits of erenumab reported in clinical trials extend to daily clinical practice.

## Key findings

- The number of patients who achieved ≥ 30% reduction in MMDs from baseline to weeks 9–12 was 195 (71.4%) of 273 patients.
- Sustained ≥ 30% reduction in MMDs at all assessment periods throughout the 52-week treatment period was achieved by 102 (34%) of 300 patients.
- Treatment discontinuation due to lack of tolerability occurred in 41 (13.7%) patients.

## Data Availability

The datasets used and/or analyzed during the current study are available from the corresponding author on reasonable request.

## Abbreviations

CI: Confidence interval
CGRP: Calcitonin gene-related peptide
ICHD-3: International Classification of Headache Disorders, 3^rd^ edition
IQR: Interquartile range
mAb: Monoclonal antibody
MHD: Monthly headache day
MMD: Monthly migraine day
MOH: Medication overuse headache
RCT: Randomized clinical trial
SD: Standard deviation
